# Cognitive control of movement via the cerebellar-recipient thalamus

**DOI:** 10.3389/fnsys.2013.00056

**Published:** 2013-10-01

**Authors:** Vincent Prevosto, Marc A. Sommer

**Affiliations:** Department of Biomedical Engineering, The Center for Cognitive Neuroscience, The Duke Institute for Brain Sciences, Duke UniversityDurham, NC, USA

**Keywords:** motor thalamus, central thalamus, thalamus, cognition, cerebellum, timing, executive control, language

## Abstract

The cognitive control of behavior was long considered to be centralized in cerebral cortex. More recently, subcortical structures such as cerebellum and basal ganglia have been implicated in cognitive functions as well. The fact that subcortico-cortical circuits for the control of movement involve the thalamus prompts the notion that activity in movement-related thalamus may also reflect elements of cognitive behavior. Yet this hypothesis has rarely been investigated. Using the pathways linking cerebellum to cerebral cortex via the thalamus as a template, we review evidence that the motor thalamus, together with movement-related central thalamus have the requisite connectivity and activity to mediate cognitive aspects of movement control.

## Introduction

The majority of our knowledge of the primate thalamus at the systems level is based on the study of circuits for sensation (e.g., retinogeniculostriate pathway). Questions of how thalamic circuits contribute to movement and cognition are largely unanswered. The more complex the behavior, the more that motor and cognitive processes will need to interact with each other. Imagine, as a brief example, the actions and calculations that are intertwined as a driver merges into highway traffic. The degree to which motor and cognitive processes may co-occur is constrained by environmental factors (Knoblich and Flach, 2001; Pulvermüller and Fadiga, 2010; Filimon et al., 2013), but it is well accepted that motor and cognitive systems must be able to share information and run simultaneously (Cisek and Kalaska, 2010; Koziol et al., 2012). Here we review evidence for cognitive processes in movement-related thalamus, with special emphasis on cognitive functions that are particularly developed in primates, as opposed to more common functions such as associative learning that are found in all vertebrates, or even arthropods (Giurfa, 2013).

**Figure 1:**
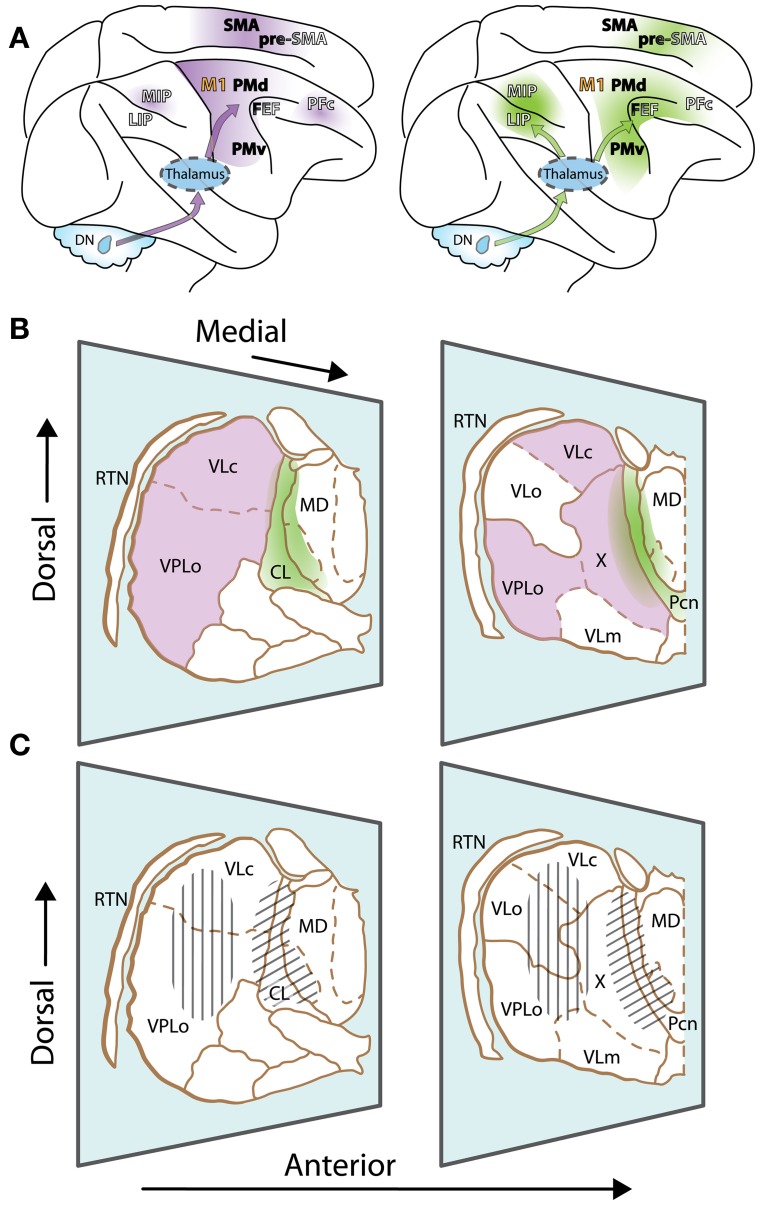
**The motor thalamus and ascending cerebellar inputs to cerebral cortex. (A)** Lateral view of the rhesus monkey brain with hemispheres separated to expose mesial wall on top. Arrows schematically represent cerebello-cortical pathways relayed via cerebellar territories in lateral (left, violet) and central thalamus (right, green). Gradients of color on cortex indicate relative strength of inputs. Left: PFc, MIP are set apart, as inputs to such non-motor cortices are relayed via medial and dorsal regions of the classic motor thalamus, topographically distinct from regions relaying inputs to motor cortices. Names of cortical areas are color-coded as follows: Primary motor cortex, orange; non-primary motor fields, black; association cortices, white (non-exhaustive presentation). FEF and Pre-SMA have dual color-coding, as they receive inputs from “non-motor” cerebellar domains, but display some non-primary motor features as well. **(B)** Two representative sections of the thalamus of *Macaca mulatta*, viewed from a lateral anterior perspective (thalamic nuclei delineated according to (Olszewski, 1952)). Only relevant nuclei are labeled. Reticular thalamic nucleus (RTN) is not part of the motor thalamus but mediates cortical inhibitory control of thalamic activity. Cerebellar domains in lateral and central thalamus are presented in violet and green, respectively. The diffuse borders of central thalamus encompass the rostral intralaminar group (central lateral nucleus, CL, and the paracentral nucleus, Pcn) and paralaminar regions of the VL complex and MD. **(C)** Same sections as **(B)**, but illustrating effector-related *functional*, as opposed to *hodological*, compartments. Hatching shows the rough somatotopic locations of regions related to arm (vertical) and eye (diagonal) movements. Other abbreviations: DN, dentate nucleus; MIP, medial intraparietal area; LIP, lateral intraparietal area; M1, primary motor cortex; PMd, dorsal premotor cortex; PMv, ventral premotor cortex; FEF, frontal eye fields; PFc, prefrontal cortex; SMA, supplementary motor area; pre-SMA, pre-supplementary motor area; VLo, ventrolateral nucleus pars oralis; VPLo, ventroposterolateral nucleus pars oralis; MD, mediodorsal nucleus; VLc, ventrolateral nucleus pars caudalis, X, nucleus X of the thalamus.

Motor thalamus is classically delineated according to cerebellar and basal ganglia projection zones. This review will primarily focus on the two juxtaposed thalamic regions that receive inputs from so-called motor and non-motor domains of the *dentate nucleus*, the output node of the lateral cerebellum. The first region corresponds to typical cerebellar territories of the motor thalamus (Figure 1A, left, and violet in Figure 1B), which are essentially found posteriorly to basal ganglia territories, in the ventral lateral complex (VL) of the thalamic nuclei (VLps and VLc subdivisions as well as nucleus X) and the oral division of the ventral posterolateral nucleus (VPLo). Those thalamic nuclei in turn project to cortical motor areas [primary motor cortex (M1), premotor cortex (PM) and the supplementary motor area (SMA)]. Additionally, projections from nucleus X and caudal regions of VLc also target the pre-SMA and frontal and parietal associative cortices (Wiesendanger and Wiesendanger, 1985a, b; Middleton and Strick, 2001; Morel et al., 2005; Prevosto et al., 2010). The second thalamic region considered in this review is composed of the central thalamus (Figure 1A, right, and green in Figure 1B). This region contains the rostral intralaminar complex [mainly the central lateral nucleus (CL) and, for cerebellar territories, to a lesser extent the paracentral nucleus (Pcn)] together with paralaminar regions of the mediodorsal nucleus (MD) and VL (Schlag-Rey and Schlag, 1989; Groenewegen and Berendse, 1994). The posterior intralaminar system (centre médian and parafascicular nuclei), heavily interconnected with basal ganglia, will not be discussed here. The central thalamus targets association cortices as well as motor cortices, with a gradient of projections (Rouiller et al., 1999; Morel et al., 2005; Prevosto et al., 2010). Most cortical regions that receive cerebellar inputs are recipients of thalamic inputs from these two contiguous thalamic regions, with different weights. As mentioned above, motor thalamus predominantly targets motor cortical regions. In contrast, central thalamus has widespread access to both associative and motor cortex.

## Contextual modulation of activity in thalamus and cerebellum

The influence of cognitive functions on the neuronal activity of motor thalamus is far from established. It is well known, however, that only a subset of neurons in the motor thalamus is concerned solely with basic motor parameters. Many of the neurons contribute, instead, to more elaborate features of movement planning and execution. This functional distinction is in agreement with findings that both cerebellum and basal ganglia are implicated in higher level functions that expand and complement their role in movement (Middleton and Strick, 1994; Aglioti, 1997; Haber and Calzavara, 2009). Similarly, motor thalamus, as classically defined by its subcortical inputs, has long been known to project to cortical regions well beyond motor and PM (e.g., Kievit and Kuypers, 1977). Conversely, top down cortical control that mediates cognitive signals from the prefrontal cortex (Brunia, 1999) may also influence movement-related activity in motor thalamus. This latter control is thought to be an important factor in volitional and selective gating of ascending inputs (Nadeau, 2008). As will be discussed below, volitional and context-dependent modulation of activity are hallmarks of cognitive influence on movement-related processing in the thalamus.

A classic demonstration that motor-related activity in thalamus is not always tightly associated with specific movement parameters came from oculomotor research (Schlag and Schlag-Rey, 1984; Schlag-Rey and Schlag, 1984). In their seminal papers, Schlag-Rey and Schlag introduced a bold proposal, namely the central controller hypothesis, which proposed that eye-movement related activity in the central thalamus specifies the timing of particular actions. The activity modulations in central thalamic regions are highly sensitive to context, and their specific sets of projections have been shown to mediate aspects of cognitive processing such as working memory (van der Werf et al., 2002). Indeed, if the context in which a movement is made influences neuronal activity during motor preparation and execution, this information must be stored and effectively accessed; in other words, working memory properties are needed (for more details on primate working memory circuits, see Constantinidis and Procyk, 2004).

Similar findings of contextually modulated activity, as well as selective modulation of preparatory activity for volitional movements, have been observed in the cerebellar dentate nucleus (Grimm and Rushmer, 1974; Mushiake and Strick, 1993; Ashmore and Sommer, 2013; Prevosto et al., 2013), the source of cerebello-thalamic projections from the lateral cerebellum. These results support the concept that some cerebello-thalamo-cortical pathways may be involved in higher order aspects of motor control and behavior. This suggestion raises two related issues. First, considering the potential involvement of cerebello-cortical circuits in cognitive functions that underlie complex behavior (Diamond, 2000; Koziol et al., 2012), what evidence exists that thalamus mediates the relevant activities? Then, for those activities that motor thalamus conveys, does the thalamus have an active, participatory role, or does it primarily act as a relay?

## Cerebello-thalamo-cortical circuits have the requisite connectivity for cognitive involvement

Although it gradually emerged that motor regions of the thalamus project to a wide array of cortical targets outside agranular (motor) cortex (Kievit and Kuypers, 1977; Wiesendanger and Wiesendanger, 1985b; Schmahmann and Pandya, 1990; Shook et al., 1991), relating those pathways to their subcortical sources has proven difficult. Indeed, beyond the confusion arising from diverse nomenclatures (Percheron et al., 1996), a structural definition of the motor thalamus has always been complicated by the fact that ascending axonal arborizations cover regions that straddle multiple cytoarchitectonically-defined nuclei (Kalil, 1981; Percheron et al., 1996; Mason et al., 2000). Conversely, thalamocortical projections originate from longitudinal regions that cross over nuclei borders (Kievit and Kuypers, 1977; Percheron et al., 1996). Therefore, the input-output arrangement of thalamic pathways is one of tremendous complexity.

Only with the advent of transneuronal tracers has it been possible to map with precision the reciprocal, polysynaptic pathways between cerebellar output nuclei and associative regions outside of the motor cortices (Lynch et al., 1994; Clower et al., 2001; Kelly and Strick, 2003; Ramnani, 2006; Strick et al., 2009; Hashimoto et al., 2010; Prevosto et al., 2010; Lu et al., 2012) (Figure 1A). It appears that the majority of cortical areas, notably prefrontal, medial frontal, and posterior parietal regions providing inputs to the cerebellum (via the pontine nuclei), in turn receive cerebello-thalamo-cortical inputs (Strick et al., 2009; Ramnani, 2012). Curiously, a number of cortical regions thought to be crucial for cognition, such as the rostral temporal lobe and the ventrolateral prefrontal cortex, do not participate in these closed loops. This fact suggests a commonality between cortical areas that communicate with the cerebellum: it appears that they all contribute to the planning, control, or monitoring of movement.

The diversity of lateral cerebellar output channels, however, raises the question of their organization. Formerly, the loops that traverse lateral cerebellum through VL (Figures 1A,B) (Asanuma et al., 1983; Stein and Glickstein, 1992) were seen primarily as pathways for posterior parietal areas to gain access to PM (Thach, 1987; Stein and Glickstein, 1992), in agreement with known VL contributions to movement planning (Strick, 1976). However, the modern understanding that cerebro-cerebellar connections are largely reciprocal, and consequently target a variety of cortical areas outside the motor cortices, forces a re-evaluation of the ways in which lateral cerebellum, and its thalamic targets, may contribute to behavior. As explained above, lateral cerebellar ascending projections may be divided largely into two streams, one relayed via motor thalamus, the other via central thalamus. It is tempting to attribute the origin of each stream to motor and non-motor domains of the dentate nucleus respectively, with corresponding motor and cognitive functions. However, while cerebellar output channels are essentially segregated from each other, many cortical areas receive inputs from both central and more lateral thalamic regions (Figures 1A,B), making it difficult to separate both streams. In the two following sections, we will attempt to illuminate how the two thalamic regions differ in their contributions to the cognitive control of movement.

## Cognitive-related inputs to central thalamus

It is notable that identified cerebellar projections to central thalamic regions (formerly “non-specific” thalamus; Sasaki et al., 1979; Kalil, 1981; Asanuma et al., 1983; Sultan et al., 2012) were first considered to be potential output pathways for cerebellar cognitive signals (Leiner et al., 1986). This hypothesis assumed that inputs relayed through the central thalamus would constitute a separate, “non-specific” pathway that would exert a general influence through widespread thalamocortical projections. This view is compatible with the fact that central thalamus targets not only association cortices but also PM, SMA, and pre-SMA with considerable divergence (Figure 1A; Morel et al., 2005). This projection system, however, has been shown to be much more specific than previously conceived (van der Werf et al., 2002) and can influence selective regions, in addition to having a general impact on cortical activation levels. Specific influences carried via central thalamus, such as the modulation of preparatory activity mentioned above, would likely have different temporal dynamics than motor-related signal carried by the motor thalamus. Functional distinction between central and motor thalamus is less obvious at the transition zone in medial and dorsal parts of VL. Indeed, the fact that cerebellar inputs to prefrontal cortex seem to be relayed via caudal VLc and nucleus X (Middleton and Strick, 2001) argue for an involvement of motor thalamic regions in higher-level functions (see below). Accordingly, it has been proposed that cerebellar-recipient neurons of the caudal regions of central thalamus may be considered part of a functional continuum with more lateral “motor” cerebellar territories (Percheron et al., 1996). It has also been suggested that the mediodorsal (MD) thalamic nucleus, the main source of thalamic inputs to prefrontal cortex (Giguere and Goldman-Rakic, 1988; Ray and Price, 1993), may convey cerebellar signals (Sasaki et al., 1979; TianLynch, 1997). If so, cerebellar inputs to MD would be expected to be found alongside motor signals from the superior colliculus (SC), which are relayed by the lateral MD to the frontal eye fields (FEF) (Sommer, 2003). Paralaminar regions of MD, however, are dominantly innervated by basal ganglia inputs, and cerebellar projections there are limited (Stanton, 1980; Kalil, 1981; Percheron et al., 1996; Mason et al., 2000; Erickson et al., 2004). It is thus likely that the majority of ascending cerebellar projections to frontal associative cortex is transmitted either via central thalamus or more lateral cerebellar territories (formerly “classical” motor thalamus).

Eye-movement related circuits show the limitations in distinguishing these pathways purely based on connectivity. The oculomotor thalamus largely overlaps with central thalamus (Schlag-Rey and Schlag, 1989; Tanaka and Kunimatsu, 2011) (Figures 1B,C, right) and targets both the lateral intraparietal area (LIP) and the FEF (Kievit and Kuypers, 1977; Huerta et al., 1986; Prevosto et al., 2010) (Figure 1A), two prominent nodes in the cortical circuits for the selection and control of eye movement. Both of these cortical regions receive inputs from the same caudal dentate region (Lynch et al., 1994; Prevosto et al., 2010). However, in comparison to LIP, dentate inputs to FEF may also be relayed via more lateral (paralaminar) thalamic regions (Okuda, 1994).

How this functional ensemble may contribute to higher level function is starting to be understood. For instance, central thalamus is known to contribute to working memory via its action on forebrain arousal (Mair et al., 2011). This action has often been related to the ascending reticular activation system, which notably provides intralaminar nuclei with profuse cholinergic inputs (Groenewegen and Berendse, 1994). Central thalamus, however, has the requisite connectivity to mediate subcortical influence on selective cortical circuits. Recent results showing that intact cerebello-thalamo-cerebral pathways are crucial for the normal functioning of working memory (Law et al., 2011) are compatible with this view.

**Figure 2:**
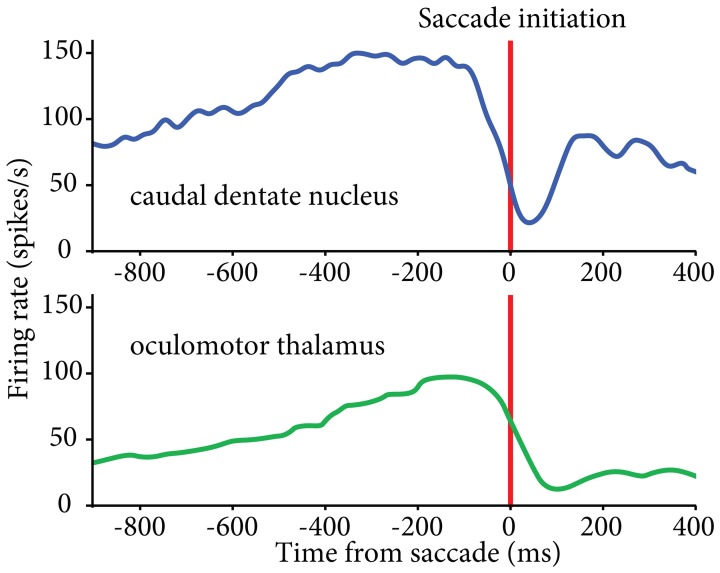
**Similar patterns of activity found in caudal dentate nucleus (top) and oculomotor thalamus (bottom) for self-initiated eye movements.** Activity aligned to the initiation of self-timed saccades. Oculomotor thalamus data reproduced from Tanaka (2007a); dentate nucleus data from our lab (see Ashmore and Sommer, 2013).

Recent data implicate the lateral cerebellum in verbal working memory, but also point out contributions to spatial processing, timing, and executive functions (Leiner et al., 1989, 1993; Chen and Desmond, 2005; Strick et al., 2009; Schmahmann, 2010; Bellebaum et al., 2012; Ramnani, 2012; Stoodley, 2012). The exact involvement of central thalamus in these functions is not yet clear, although there is evidence that it contributes to timing, in addition to working memory. Saccade-related neurons in central thalamus have been shown to display early activity that is particularly associated with the timing of self-initiated eye movements (Tanaka, 2007a; Tanaka and Kunimatsu, 2011). Complementary saccade-related activity patterns have been found in central thalamus that could signal the timing for acquisition and processing of reafferent information following saccades (Schlag-Rey and Schlag, 1984). Although neuronal activity related to self-initiated eye movements has been observed in basal ganglia, the thalamic neurons related to the timing of proactive movements were found predominantly in cerebellar territory (Tanaka, 2007a), in agreement with the putative involvement of the dentate nucleus in the initiation of volitional movements (Shibasaki et al., 1986; Ashmore and Sommer, 2013) (Figure 2). Thus, central thalamus appears well suited to transmit anticipatory activity related to volitional, self-timed movements from the lateral cerebellum to connected cortical areas (Maimon and Assad, 2006; Fried et al., 2011).

## Cognitive-related inputs to VL/VPLo thalamus

While encouraging, the above conclusions were based largely on oculomotor studies. An important issue is the extent to which those findings generalize to skeletomotor movements, which are associated with thalamic activity in the VL and VPLo nuclei (Figures 1B,C, left).

Recordings in the cerebellar dentate nucleus (a dominant contributor to VL, see above) have described a population of neurons with long-lead activity (Grimm and Rushmer, 1974; Strick, 1983). Correspondingly, among arm-movement VL units, most cerebellar-recipient neurons increase their discharge before movement initiation, sometimes before the first change in electromyographic potential (Strick, 1976; Anderson and Turner, 1991). While the cerebellum is known for its role in the timing of movement (Salman, 2002), the involvement of cerebellar-recipient motor thalamus in the timing of volitional arm-movement is less clear. Notably, arm-movement neurons in cerebellar-recipient thalamus are commonly found to be more responsive to visually cued movement than to spontaneous or memory-based movement (van Donkelaar et al., 1999). Similar contextual modulation has been described in neurons from the “motor” domain of the dentate that target ventral premotor cortex (Mushiake and Strick, 1993) as well as in the medial intraparietal area (Colby and Duhamel, 1996), a region of the posterior parietal cortex that receives dentate inputs via motor thalamus (Prevosto et al., 2010). However, comparable effects have been described across effectors and brain structures, as exemplified by greater response to visually-guided than to spontaneous arm movements (van Donkelaar et al., 1999, 2000) and saccades (Mano et al., 1996) in thalamus and cerebellum, respectively. Thus, context dependency of neuronal activity may reflect a widespread influence found across functional divisions of the thalamus. Notably, a growing body of imaging and clinical studies (Ide and Li, 2011; Peterburs et al., 2011; van der Salm et al., 2013) indicate that cerebellar territories of the motor thalamus provide a critical contribution to executive control functions of the frontal lobe. This contribution could rely on motor thalamic inputs to non-primary motor regions and (less densely) to associative cortical regions, or central thalamic inputs to the same regions, or both (Figure 1A).

More speculative is the potential role of motor thalamus in coordinating cognitive and motor aspects of language production. Results from stimulation studies found that language deficits can be induced at the same thalamic location as motor effects related to language production (Johnson and Ojemann, 2000). This intriguing finding is in agreement with the demonstration that ventral premotor cortex contains neurons specifically activated during vocalization (Coudé et al., 2011), in a region that receives dense projections from cerebellar-recipient thalamus (Matelli et al., 1989). This ventral premotor region is considered homologous to the motor portion of Broca's area in humans (Binkofski and Buccino, 2004) and is part of a dual cerebello-cortical system supporting verbal working memory (Chen and Desmond, 2005).

## The thalamus as a site for motor/cognitive interactions

If the motor and central thalamic neurons are involved in higher-order functions, the question remains whether their activities are driven mainly by their ascending inputs (i.e., whether thalamic neurons are just relays), or if these inputs, coupled with descending cortical modulation, results in thalamus-specific information processing.

Evidence from the well-characterized circuit from the SC to FEF via MD thalamus mentioned above (Sommer and Wurtz, 2004a, b) offers a template for a relay function: SC-receiving MD relay neurons essentially behave as a high-pass filtered version of SC inputs (Sommer and Wurtz, 2004a). This is fitting with the role of this circuit as a corollary-discharge pathway, i.e., carrying copies of a motor command.

Cerebellar-receiving thalamic neurons, which are not necessarily part of a corollary-discharge pathway, may behave differently. It is known that the activity of motor thalamic neurons is shaped by cortical inputs (Guillery, 1995). This is evidenced, for example, by the high baseline firing rate of pallidal-recipient thalamic neurons, which likely results from a dual modulatory cortical control, one direct and excitatory, the other indirect and inhibitory (Selemon and Goldman-Rakic, 1988; Anderson and Turner, 1991; Guillery, 1995; Band and van Boxtel, 1999). Similarly, the activity of eye position thalamic neurons reflects properties of both brainstem inputs (separate horizontal/vertical channels; delays compatible with ascending inputs) and cortical inputs (hysteresis; long lead activity) (Schlag and Schlag-Rey, 1984; Tanaka, 2007b), in agreement with the view of intralaminar nuclei as a site of convergence of subcortical and cortical inputs (Kemp and Powell, 1971). It is thus conceivable that cortical inputs modulate cerebellar-recipient neurons' activity at least as strongly as their primary drive.

Another type of thalamic-specific interaction potentially occurs through converging ascending inputs from multiple subcortical sources. Demonstrated convergence patterns of this type, such as between dentate and interpositus nucleus projections (Shinoda et al., 1985), or cerebellar and basal ganglia projections (Sakai et al., 2002), have been studied only within pathways contributing to motor cortical areas, and are essentially inconclusive for the question of motor-cognitive interaction in the thalamus. However, the thalamus also has been shown to convey cerebellar inputs to striatum that derive from both motor and non-motor regions of the dentate nucleus (Kemp and Powell, 1971; Hoshi et al., 2005). Interestingly, the central thalamus seems to be the main relay for this pathway (Ichinohe et al., 2000; Hoshi et al., 2005). It is conceivable that, reciprocally, basal ganglia inputs to cerebellar thalamic regions contribute to both motor and non-motor circuits.

The two preceding types of interactions (subcortico-cortical and subcortico-subcortical) point to a dominant role of central thalamus in mediating cognitive aspects of movement control. Another aspect of thalamic connectivity suggest a third way by which both motor a central thalamic regions could actively contribute to cognitive control of movement. There is evidence that single thalamic regions provide inputs to functionally separate cortical areas, such as motor and associative cortices (Wannier et al., 1992). This divergence seems to represent a final sorting of signals that arise from selective regions of the dentate nucleus. Data from separate studies suggest that dentate regions where output channels overlap could target distributed cerebral cortical regions. Such is the case for caudal dentate projections to the FEF and LIP (Lynch et al., 1994; Prevosto et al., 2010), for dentate projections to the anterior intraparietal area, PMv, and M1 (Clower et al., 2005), and possibly for ventral dentate to the medial intraparietal area and the pre-SMA (Wiesendanger and Wiesendanger, 1985a; Akkal et al., 2007; Prevosto et al., 2010). While this organization does not validate a specific role for the thalamus in motor-cognitive interaction, it indicates that there may be a unique and overlooked role of motor thalamus in conveying such signals to separate regions. How those dentate projections interact in the thalamus with top-down cortical control and ascending inputs from other subcortical regions is not known, although it can be construed that this arrangement would likely contribute to behavioral flexibility.

## Conclusions

Concordant results from a variety of studies indicate that movement-related thalamus takes part in circuits for higher-level control of behavior. A prior, parsimonious viewpoint was that distinct subcortico-thalamo-cortical pathways were likely to mediate separate functions (e.g., Sommer, 2003). Recent findings seem to paint a more nuanced picture.

First, each pathway consists of sub-streams, both in the thalamus and in the subcortical networks leading to it. These sub-streams may play differing roles in movement, or perhaps have differing contributions according to behavioral context. Accordingly, cerebellar-receiving thalamic neurons are possibly involved in both straightforward visuomotor control and higher level modulations of that control, such as the initiation of self-timed movements (Figure 2). The degree to which these conclusions hold across effectors, however, is still unclear. Second, the discovery of reciprocal, disynaptic connections between the cerebellum and the basal ganglia (Bostan and Strick, 2010) imply direct communications between these two principal pathways to cerebral cortex. The fact the central thalamus is posited as the main relay for cerebellar inputs to striatum underlines its relevance for high-level behavior in association with “core” motor thalamus.

Hence the overall conclusion from clinical, physiological, and anatomical studies is that the thalamus appears suited to relay, or perhaps even to mediate, the influence of cognitive processes on motor processes. Because in mammals, and particularly in primates, most behaviors comprise a cognitive component, it is not surprising to find prevalent cognitive modulation of motor circuits. The surprise comes perhaps from the fact that circuits beyond the cerebral cortex, including nuclei of the motor and central thalamus, seem to be so critical for cognitive-motor interactions.

### Conflict of interest statement

The authors declare that the research was conducted in the absence of any commercial or financial relationships that could be construed as a potential conflict of interest.
